# An overview of the CellML API and its implementation

**DOI:** 10.1186/1471-2105-11-178

**Published:** 2010-04-08

**Authors:** Andrew K Miller, Justin Marsh, Adam Reeve, Alan Garny, Randall Britten, Matt Halstead, Jonathan Cooper, David P Nickerson, Poul F Nielsen

**Affiliations:** 1 Auckland Bioengineering Institute, The University of Auckland, Auckland, NZ; 2 Department of Physiology, Anatomy and Genetics, Sherrington Building, Parks Road, Oxford OX1 3PT, UK; 3 Oxford University Computing Laboratory, Wolfson Building, Parks Road, Oxford OX1 3QD, UK; 4 Department of Engineering Science, The University of Auckland, Auckland, NZ

## Abstract

**Background:**

CellML is an XML based language for representing mathematical models, in a machine-independent form which is suitable for their exchange between different authors, and for archival in a model repository. Allowing for the exchange and archival of models in a computer readable form is a key strategic goal in bioinformatics, because of the associated improvements in scientific record accuracy, the faster iterative process of scientific development, and the ability to combine models into large integrative models.

However, for CellML models to be useful, tools which can process them correctly are needed. Due to some of the more complex features present in CellML models, such as imports, developing code *ab initio *to correctly process models can be an onerous task. For this reason, there is a clear and pressing need for an application programming interface (API), and a good implementation of that API, upon which tools can base their support for CellML.

**Results:**

We developed an API which allows the information in CellML models to be retrieved and/or modified. We also developed a series of optional extension APIs, for tasks such as simplifying the handling of connections between variables, dealing with physical units, validating models, and translating models into different procedural languages.

We have also provided a Free/Open Source implementation of this application programming interface, optimised to achieve good performance.

**Conclusions:**

Tools have been developed using the API which are mature enough for widespread use. The API has the potential to accelerate the development of additional tools capable of processing CellML, and ultimately lead to an increased level of sharing of mathematical model descriptions.

## Background

Systems of differential algebraic equations (DAEs) [1] are one particularly common and useful form of mathematical model. These systems are of the general form

where *F *is a function, *t *is the independent variable, **x **is the vector of state variables, and **x' **is the vector of derivatives of the state variables.

DAE systems are often broken up into individual equations, each of which hold true. Systems of DAEs are used to model a wide variety of biological processes, across a diversity of scales. For example, at one extreme there are models describing the action of ion channels [2], and at another extreme, models of predator-prey dynamics [3].

Historically, models of DAEs have been exchanged and archived by publishing equations, constant values, initial conditions, specific protocols, and other associated information in a scientific paper. Someone wanting to independently compute results from the published model then needs to convert that model back into a computer program. This process is both time-consuming and error prone. Reviewers are unlikely to check that the published model accurately corresponds to numerical results presented in the paper.

Likewise, it becomes prohibitively expensive to do integrative biology [4], as building a large model out of several pieces then requires significant effort on each of the pieces already in the literature.

CellML [5] is an XML [6] based format for representing mathematical models, capable of representing DAE systems (as well as other mathematical relationships). As such, it provides an ideal mechanism for the exchange and archival of models. There are public databases containing large numbers of CellML models, such as the CellML Model Repository [7]. The BioModels database [8] also provides models which have been translated from Systems Biology Markup Language (SBML) to CellML.

However, for the scientific advantages of using formats such as CellML and SBML for mathematical model exchange to be fully realised, it is important that software used by modellers is able to read and write models in these formats. It is also important that the scientific community has the ability to easily develop software which relies on the existing databases of models in these formats.

Using an Application Programming Interface (API) simplifies the task of processing an XML language, thus APIs are important to the exchange of information.

Supporting CellML correctly can be a difficult task, due to some of the more complex features in the CellML language. It is therefore important that software developers do not need to re-invent the same functionality every time they develop a new tool. We thus present both an API for working with CellML models, and an efficient implementation of that API.

SBML [9] is another XML-based format, used for encoding computational models of biochemical reaction networks. It requires that implementations support elements specific to biochemical reaction networks (although it also provides more general mechanisms for representing models), and thus differs from CellML in that CellML avoids domain-specific elements. There is an API for processing SBML models, known as libSBML [10]. The CellML API serves an analogous purpose to libSBML, except for CellML rather than SBML models.

## Implementation

The CellML API is a platform and programming language independent description of interfaces, with attributes and operations on the interfaces. These attributes and operations are used to retrieve information about the model, or alternatively to manipulate the model in memory.

**Figure 1:**
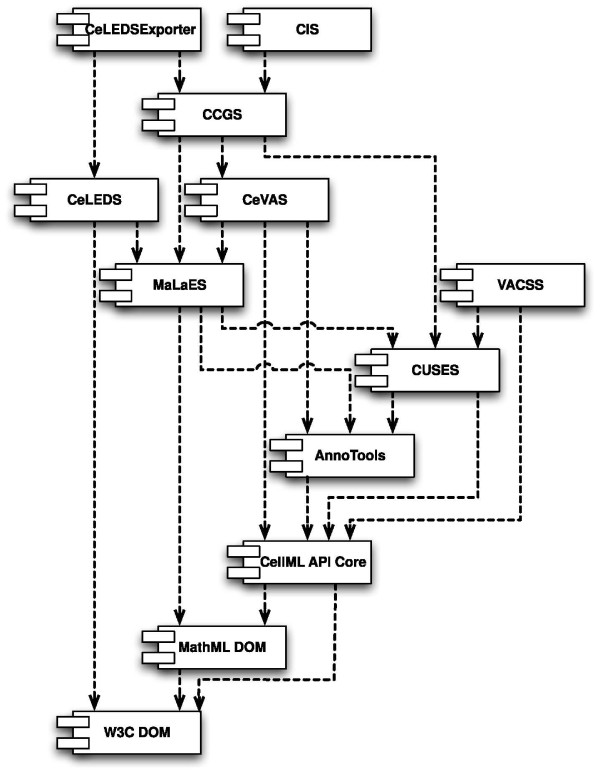
**UML Component diagram of the CellML API**. This standard UML Component Diagram [43] shows the dependencies between the different components of the CellML API. This diagram applies equally well to the dependencies between the interfaces, and the dependencies required by our implementation. Some indirect dependencies have been omitted for clarity.

The overall architecture of the API consists of a core API, along with a series of extension APIs (see Figure 1). The extension APIs are listed here, and discussed in detail later:

• Annotation Tools Service

• CellML Variable Association Service

• CellML Units Simplification and Expansion Service

• Validation Against CellML Specification Service

• MathML Language Expression Service

• CellML Code Generation Service

• CellML Language Export Definition Service

• CellML Integration Service.

The extension API implementations are cleanly separated from our implementation of the core API, so that alternative implementations are possible.

The API is specified using OMG IDL [11], and is made available under an Open Source/Free license, at http://www.cellml.org/tools/api/. It is suitable for both CellML 1.0 [12] and CellML 1.1 [13] documents. All attributes and operations in the IDL files are documented in place using the Doxygen comment format [14]. The choice of this programming language independent format to specify interfaces makes it possible to define bindings to the API from many programming languages. We have developed bindings for C++ [15], Java [16], and JavaScript (via XPCOM) [17].

In addition, we have developed an implementation of the API, optimised to reduce time taken to run a test-suite of typical tasks. This implementation is written in C++, and is based on the libxml2 XML parsing library [18], and our own implementation of the W3C DOM [19] and MathML DOM [20]. Language bindings and bridges offer access to the API from C++, Java, JavaScript, and from an even wider range of languages (for example, Python [21]) over CORBA [11].

## Results

We firstly discuss the basic functionality used throughout the core API. We then discuss how the API can be used to process CellML Metadata and imports. We discuss the object model and memory management scheme used by the API, and proceed to discuss each of the extension APIs in sequence. We then discuss our suite for testing API implementations, and conclude the section with a comparison to other software with similar functionality.

### Core API

The scope of the core API is the basic manipulation of, and access to, the content of CellML models. The facilities for information retrieval in the API are closely aligned to the arrangement of XML elements in a CellML document. The IDL specification for the core API can be found in the file interfaces/CellML APISPEC.idl, in the CellML API source tree.

There is one object for each CellML element in the document. These objects implement an interface, which is specific to the type of the CellML element. The interfaces of these elements all inherit (directly or indirectly) from the CellMLElement interface. This interface provides functionality which is useful on all elements. For example, it provides the ability to insert or remove any of the child elements of the element concerned, and to set temporary user data annotations, identified by a unique key, against the elements. These annotations do not form part of the in-memory DOM representation, and so do not, for example, appear in the generated XML when the model is serialised.

The interfaces for CellML elements which have a mandatory name attribute all inherit from the NamedCellMLElement interface. This interface provides a name attribute (which can be fetched or set), and inherits from the CellMLElement interface. For example, CellMLComponent inherits from NamedCellMLElement, because the CellML specifications require that all component elements have a name attribute.

For each type of child CellML element allowed by the CellML specification, the interface for the parent element has a read-only attribute for retrieving all the CellMLElements of that type. The returned set implements an interface specific to the type of element expected. For example, component elements can contain variable elements, so the CellMLComponent interface has an attribute called variables, of type CellMLVariableSet.

These specific types of set follow an inheritance hierarchy parallel to those of the element objects in the set. Each set interface has a corresponding iterator interface, which allows each object to be fetched in sequence. Because the iterator interface is specific to the object being fetched, the required interface is returned, avoiding the need to call QueryInterface (see the section on the object model and memory management for information on QueryInterface). However, it is also possible to use the less specific (ancestors in the interface inheritance hierarchy) set interfaces to retrieve a less specific (ancestor) iterator object (for example, for use in generic code which works on more than one type of element).

Iterators derived from NamedCellMLElementIterator also provide interfaces for fetching elements by name. All descendant iterator interfaces provide more specific fetch by name operations.

Set interfaces also provide facilities for modifying the relevant sets by inserting CellML elements. Because order is not important to the meaning of the model, the iteration and insertion facilities provide no control over the actual order of the elements in the model.

CellML makes heavy use of the namespace facilities in XML [22]. CellML provides for extension elements, that is, elements which are not in the usual CellML, MathML, or RDF namespaces. The CellMLElement interface provides an attribute extensionElements, of type ExtensionElementList. The ExtensionElementList interface allows for DOM elements which are not in the CellML namespace to be examined and manipulated.

### Handling CellML Metadata

In addition, CellML models commonly contain metadata [23] encoded in RDF/XML [24]. There are many different ways to process the RDF data encoded in RDF/XML. The Model interface provides an operation called getRDFRepresentation, which takes a type URI to describe the type of RDF representation requested. These representations provide an interface deriving from the RDFRepresentation interface. The API requires that all implementations provide at least an implementation which provides a serialised RDF/XML document as a string, and an implementation which provides a DOM Document node for the data as a single RDF/XML. In order to produce these RDF/XML outputs, implementations need to pool several different fragments of RDF found throughout the document into a single RDF/XML document. Applications can also modify the RDF/XML and push it back into the model.

Our implementation of the CellML API also provides an interface allowing access to and modification of the RDF triples [25] found in the model.

### Dealing with model imports in CellML 1.1

CellML 1.1 provides for components and physical units to be imported into models from other models [13]. An import is created by adding an import element, which refers to another model to be imported by URI. The import element has child elements which describe which components and units from the imported model are accessible in the importing model. The CellML API provides facilities to allow such information to be accessed.

The result of supporting CellML 1.1 is that processing one mathematical model can require that more than one CellML file be examined. To deal with this issue, the API introduces the following two concepts: an imported model is said to be instantiated once it has been loaded. When all imports required for a mathematical model have been loaded (including models which are imported by an imported model), the model is said to be fully instantiated.

The API has an operation for selectively instantiating particular imports, as well as an operation for fully instantiating the model. For imports that are instantiated, the model element is also accessible, as well as the components they import.

We have included three separate attributes for sets of components in the model, with three corresponding sets of units:

• local component set - contains only the components in the particular CellML file (excluding imported components);

• model set - contains all components which are in the local set, and also the import component elements (that is, the component element children of import elements, describing which components are imported) in the same file; and

• full set - contains all components in the model, across all files making up the model. Where the model containing a component is uninstantiated, the import component element is provided by iterators. When a model is instantiated, the components in the imported model are returned by the iterators, and in addition, these models are examined to identify further imported models to search for components, as appropriate.

The three corresponding sets of units follow the exact same semantics as the sets of components, except over units rather than components.

### Some technical details

The interfaces defined in the API all use the inheritance capabilities of OMG IDL to derive from a base interface, called IObject. IObject is modelled after the similarly named interface in the XPCOM object model. The IObject interface is used to provide interfaces for basic common facilities relating to the object underlying the interface, such as maintaining the reference count (as discussed later), and providing a unique identifier for each object. This unique identifier is useful for determining if two interface references describe the same object, and for building data structures which require that objects can be compared. API implementations use reference counting [26] to determine when there are no remaining references to a particular object. The IObject interface has operations for incrementing and decrementing the reference count. In order to ensure reference counting works correctly, a few simple rules are followed consistently throughout the API design (and the API design relies on the same rules being followed by code which deals with the API). All operations and attributes which provide an interface reference also increase the reference count of the underlying object. For example, in the case where the operation creates a new object, but no internal references to the new object are kept, the reference count of the returned interface should be one. Secondly, for every time the reference count is incremented by invoking the add_ref operation (or by obtaining a returned interface), the programmer must ensure that eventually, the reference count is decremented by invoking the release_ref operation. The API is designed to be accessed through wrappers, and so the actual storage of the object may even reside on a different machine to the wrapper providing the interface being used. For this reason, the third rule arises: add_ref invocations must always be matched with a release_ref invocation on the exact same interface pointer (as opposed to a different interface pointer for the same object, which may point to a different wrapper around the same object).

It is worth noting that while IObject provides facilities for reference counting, many programming languages perform automatic garbage collection. When using a direct bridge to these languages, the wrapper code will automatically call add_ref and release_ref on behalf of the user, and so the need for explicit memory management is avoided. For example, the Java bridge makes use of the finalisation facilities in Java, combined with the memory management facilities provided by the Java Native Interface, so that Java users do not need to explicitly modify reference counts.

In addition to the reference counting scheme, the IObject interface also provides a QueryInterface operation. This operation is used to ask an object if it supports a particular interface, and if it does, to provide an interface representation. As discussed earlier, the API is often accessed through wrapper code, and so users of the API should always perform QueryInterface operations on API interfaces, rather than directly using the language-specific casting mechanisms.

Objects which are created by API implementations exist purely in memory (whether the model was, for instance, created *ab initio*, or loaded from a file). Modifications can be made to the model in memory. The original file will only be updated if the application uses the API to serialise the CellML model back to XML, and then writes that XML to disk, replacing the original file. Likewise, if the same model is loaded twice, there will be two separate, and independent instances of the model in memory. Modifying one instance will not automatically change the other instance. Where a model is imported, a separate instance of the imported model exists for each import element, and for each instance of the importing model. However, all elements, sets, and iterators in the core CellML API are 'live', in the sense that making any change to an in-memory instance of a model through the API will immediately affect responses from the API, even if the element, set or iterator was retrieved prior to when the change was made. For example, if an iterator is created, and has iterated through all units elements but one in a model, and that remaining units element is deleted, and next element is retrieved from the iterator, it will return a null value, signifying that there are no remaining units elements to iterate.

The API makes use of the exception mechanism in OMG IDL to handle exceptional conditions (for example, when the API cannot perform the requested operation, because of element structure which is inconsistent with the CellML specification). Our implementation makes consistent use of exception safety techniques [27], such as the Resource Acquisition Is Initialisation (RAII) pattern, to ensure that memory leaks do not occur when exceptions are raised.

The language independent IDL based interfaces do not provide a solution to the 'bootstrap' problem of how an interface is initially obtained, for example, the interface for creating a new model. The solution to this problem is language dependent. In each language, we provide functionality to retrieve a pointer to a bootstrap interface. For example, in C++, this is obtained by a method defined in a header. The bootstrap interface is defined in IDL, and therefore standardised across all language bindings. Each extension API has a separate bootstrap interface.

Our implementation of the API is not designed to allow for two writes (for example, a modifying operation, or use of an attribute setter) to occur concurrently, or for a read to occur concurrently with a write, on the same model. Applications accessing the same model on multiple threads need to either protect all access to the API with a mutex, or more efficiently, use a read-write lock to ensure that there is no activity concurrent with a write.

### Extension APIs

In addition to the core API, we have also produced APIs to provide services which are beyond the scope of the core API.

The core API does not depend upon the extensions, and so individual API implementations can choose not to support all extension APIs. However, all extensions depend upon the core API, and some extensions also depend on other extensions (see figure 1).

### The Annotation Tools Service

The core CellML API provides basic facilities for in-memory annotation of elements in the CellML model with arbitrary user-supplied objects. However, the user data annotations are difficult to use for some applications, because the core API requires that user data be associated with a key, which must be manually removed when the application has finished with it.

The Annotation Tools (AnnoTools) API provides the ability to allocate and release a set of annotations, without needing to worry about interfering with other annotations being placed by independent calls to the same code, or about needing to individually remove all annotations left on objects.

The IDL specification for the AnnoTools API can be found in the file interfaces/AnnoTools.idl, in the CellML API source tree.

AnnoTools implementations generate a unique prefix for each AnnotationSet, and allow the user to set annotations with that prefix. They keep an internal list of all annotations which were added, and clear all annotations in the AnnotationSet when the AnnotationSet is destroyed.

The AnnoTools API also includes facilities for more easily setting and retrieving string, integer, and floating point annotations.

### The CellML Variable Association Service

The CellML Variable Association Service (CeVAS) API facilitates the treatment of interconnected CellML variables as the same mathematical variable (albeit possibly in different units). These variables may come from different components, some of which may be imported from different models.

The IDL specification for the CeVAS API can be found in the file interfaces/CeVAS.idl, in the CellML API source tree.

CellML 1.0 and 1.1 require that variables which are connected to variables in other components have a public or private interface value of 'in' or 'out'. Whether the public or private interface applies depends on the encapsulation relationship between the components. In CellML, all 'in' interfaces must be connected to an 'out' interface, encapsulation is always acyclic, and valid CellML models have a finite number of variable elements. This means that, in a complete and valid model, there is always a variable in each connected network of variables that has no 'in' interfaces. This variable is called the source variable, and is used by CeVAS as a representative of all variables connected (directly or indirectly) to it.

The interface allows users to supply a CellML Model interface, and pre-compute which variables are connected. All variables connected to a particular variable can be iterated, and the source variable can be retrieved.

This is implemented using an efficient disjoint sets algorithm, which allows for inverse Ackerman amortised time merges of sets [28]. Initially, every variable in the model is treated as a set of size 1. The algorithm iteratively processes all connections in the model, merging the disjoint sets associated with each of the two connected variables. Therefore, the amortised time complexity of processing a model with *n *components and *m *connections is in *O*(*nα*(*m*, *n*)), where *α *is the inverse Ackerman function. Note that the inverse Ackerman function grows very slowly; for example, *α*(2, 2) = 1, while *α*(10^20^, 10^20^) = 3.

### The CellML Units Simplification and Expansion Service

The CellML Units Simplification and Expansion Service (CUSES) API provides facilities for processing physical units in a CellML model.

The IDL specification for the CUSES API can be found in the file interfaces/CUSES.idl, in the CellML API source tree.

CellML has a set of built-in units. These units are defined in terms of the SI [29] base units; ampere, candela, kelvin, kilogram, metre, mole, and second. Other pre-defined units are defined in terms of these. For example, the Joule is defined as k*g*.m^2^.s^-1^. In addition, the modeller can define their own derived units (for example, mmol/L for concentrations), or a new base unit. However, when processing models, it is important to know what the relationship between connected variables is, so the appropriate conversions can be performed, if necessary. For example, when a variable in metres is connected to a variable in millimetres, tools are expected to insert an implicit conversion factor, so the same variable is compatible across the two components. CUSES allows tools to implement this more simply.

All units are firstly expanded to be expressions in terms of the base units. SI Prefixes are converted to multipliers. All units are converted to a canonical form, consisting of the product of powers of base units, each base unit occurring at most once, possibly with a single multiplier and/or offset. The base units, and their corresponding exponents, are exposed to users of the API in an enumerable list of base unit instances. Facilities are provided to enquire whether two units are dimensionally equivalent. This is useful for determining if a connection is valid. The necessary offset and multiplier needed to perform a conversion can also be retrieved.

### The Validation Against CellML Specification Service

The Validation Against CellML Specification Service (VACSS) API accepts files which are putatively CellML files, and identifies whether or not the CellML is valid, and where the file is not valid, it attempts to build a list of the problems.

The IDL specification for the VACSS API can be found in the file interfaces/VACSS.idl, in the CellML API source tree.

Errors that can be detected fall into two types:

• representational - errors relating to the encoding of CellML in XML, such as essential elements or attributes which are missing, or illegal extraneous elements; and

• semantic - higher-level errors, where the basic elements of the CellML are in the correct form, but there are inconsistencies, such as references to names which are required to exist but do not, or violations of any of the numerous rules specified in the CellML Specification. Semantic warnings, such as about potential units problems in mathematical equations, due to dimensional inconsistency, are also made available.

### MathML Language Expression Service

One task which is common to many applications is to convert the fragments of MathML embedded in CellML documents into fragments of text in some other linear text-based representation, such as programming language source code.

The MathML Language Expression Service (MaLaES) API provides functionality to assist with this task. MaLaES makes use of CeVAS in order to identify the source variable corresponding to each MathML ci element (*i.e. *reference to a variable by identifier). It then makes use of AnnoTools to retrieve an annotation (which the user can set) containing the symbol to be used for that variable in the output. The IDL specification for the MaLaES API can be found in the file interfaces/MaLaES.idl, in the CellML API source tree.

It is often the case that these transformations need to take units into account, to ensure that all variables in the MathML contain any necessary conversion factors. MaLaES thus allows variables (referenced by ci) to be converted into the units of the source variable, and also for the result of an expression to be converted. In order to allow for conversion into many different languages to occur, MaLaES uses a specification in a custom format called MAL (MathML to Language). The MAL description describes the mapping between MathML elements and their forms in the output text-based representation (allowing for pre-order, in-order, or post-order traversal of the MathML expression tree, with arbitrary separators between arguments), as well as describing the precedence of each operation, what strings are used to begin and end groupings of low precedence operators inside a higher precedence operator, and the format of conversions. The MAL is precompiled into an efficient in-memory representation, which can then be used to generate output.

### The CellML Code Generation Service

Another common task is to convert an entire CellML model into code in a procedural programming language, capable of solving the model. The CellML Code Generation Service (CCGS) API simplifies this task.

The IDL specification for the CCGS API can be found in the file interfaces/CCGS.idl, in the CellML API source tree.

The CCGS is specialised for the common case where there is a single independent variable (in many models, time) and the index of the DAE system is at most one [30,1]. Users of the API obtain the CodeGeneratorBootstrap interface pointer through the language specific bootstrap process, and then use the createCodeGenerator operation to obtain a CodeGenerator interface.

On this CodeGenerator interface, it is possible to specify a wide range of different attributes about the language to be generated. This means that code can be generated for a wide range of procedural programming languages (in some cases, with a requirement for some post-processing to fold long lines or perform similar transformations).

Because CCGS relies upon MaLaES to translate individual mathematical expressions into the correct text-based form, the user also needs to supply a MAL description for the language of interest.

CCGS uses the terminology 'computation target' (represented by a ComputationTarget interface pointer) to represent anything which is required to be computed to evaluate the equations in a CellML model (including those with a constant value, in which case computation is merely assignment to that value). There is not a one-to-one relationship between variables in the CellML model and computation targets. For example, there may be a variable called *x*, with an initial value of 0, and then an equation such as . In this case, *x *and  are both computation targets (*t*, the independent variable, is also treated as a computation target for consistency). Note that when a variable is used in several components, but the variables are connected together (making them the same mathematical variable), there will only be one computation target for all the variable elements.

CCGS gives every computation target a degree (degree zero means that it is the original variable, degree one means it is the first derivative of the variable, degree 2 means it is the second derivative, and so on). All computation targets which have a corresponding computation target of higher degree are treated as being state variables, while computation targets with a lower degree computation target are treated as being rates. Computation targets which have both a higher and lower degree computation target are in the unique position of being both a rate and a state variable. CCGS generates code for this case by making use of the standard technique for transforming an ordinary differential equation (ODE) system with higher derivatives into an equivalent ODE system with no higher than first order derivatives [31].

Initially, CellML variables which are in fact constant in value are identified and marked as such. Variables which are computable using only constants are in turn classified as constants (with this process repeating until no further constants can be identified). It is possible that a system of simultaneous equations may need to be solved in order to determine *n *otherwise unknown constants from *n *equations (but when it is possible to split this into small subsystems, it is more efficient to do so). This is done in our implementation using a heuristic algorithm which guarantees that the smallest possible systems are found when the largest indivisible system needed to be solved has at most three equations with three unknowns, and has given good results in our testing.

After identifying the order in which equations or systems need to be solved, code is generated for them. This is done using one of three different patterns supplied to CCGS by the application. Where CCGS needs to compute a computation target *y *using an equation like *y *= *f *(*x*_1_, *x*_2_, ..., *x*_*n*_), CCGS will use the assignment pattern to directly assign into the symbol for *y*. In other cases, the equation might be in the form *f *(*y*, *x*_1_, *x*_2_, *x*_3_, ..., *x*_*n*_) = *g*(*y*, *x*_1_, *x*_2_, *x*_3_, ..., *x*_*n*_), in which case the univariate solve pattern is used to compute *y*. Finally, for systems of equations, the multivariate solve pattern is used.

Any computation targets which are not constants, states, nor are rates, are classified as being 'algebraic computation targets'. In the same fashion as is done for constants, CCGS works out a directed acyclic graph for the order in which systems or equations are used to work out the rate and algebraic computation targets, using the constants, states, and the independent variables. However, these computations are split into two code fragments. The first code fragment contains all computations necessary to compute the rates from the states, constants, and independent variables, while the second code fragment computes any remaining algebraic computation targets not computed in the first code fragment. This separation allows for more efficient processing of models, because at many time steps, the integrator may not want to report back any results, and so there is no need to evaluate computation targets that are not required to compute the next time step.

CCGS has the capability to automatically assign indices into four different arrays:

• constants array - stores the values of any computation targets which do not depend on the independent variable, or upon any of the rate or state computation targets;

• states array - used to store the values of each state computation target;

• rates - used to store the values of the rate of change corresponding to each state computation target; and,

• algebraic array - used for all remaining variables.

The CodeGenerator object allows the first index to be assigned in each array to be set (for example, to be 0 in languages like C [32] where array indices start at 0, and 1 in other languages like MATLAB). In addition, the user can supply a pattern, for example STATES [%], to describe how the arrays are dereferenced in the output programming language. The caller can also supply their own AnnoSet object, and explicitly provide a name for each computation target if this is required.

Overall, four different code fragments are available. Firstly, the fragment to initialise constants, as discussed above. Secondly, the fragment to compute the rates (and all algebraic computation targets needed to compute these rates). Thirdly, the fragment for the remaining variables. The final code fragment contains any functions which needed to be generated (using a pattern supplied to the CCGS) in order to evaluate the code. These functions can then be called from the univariate and multivariate solver patterns, and also in MAL specifications, such as those for evaluating definite integrals.

As a CCGS implementation processes models, it will also check for and report back on certain error conditions, such as models which have extraneous equations (reported as being overconstrained), or models which have too few equations to compute all computation targets (i.e. underconstrained models). As CCGS only supports DAEs of index one or lower, it will, for example, report that the model is incorrectly constrained if the model is a valid index two DAE.

### The CellML Language Export Definition Service

Defining a new programming language for use with MaLaES requires setting up a MAL description of the language, and configuring this through the API. However, it is convenient to be able to exchange this information with other users, in order to allow for the definition of arbitrary languages by the user. The CellML Language Export Definition Service (CeLEDS) allows for the MAL description of a language to be embedded in an XML file. In addition, it provides a generalised dictionary service, to allow information required to generate output for different languages to be provided to the consumer of the CeLEDS service. The IDL specification for the CeLEDS API can be found in the file interfaces/CeLEDS.idl, in the CellML API source tree.

The CeLEDSExporter service builds upon that offered by CeLEDS to support full code generation (based upon CCGS). Instead of programmatically setting attributes on the CodeGenerator interface, all information is specified in a standardised XML format, along with the MAL description. In addition, CeLEDS contains information on the super-structure of the program, including unchanging fragments of code which are required to allow the program to run (such as any supplementary function definitions).

This means that all the information required to generate code for a language is encapsulated in a single XML file, which can be read in at run-time. Users can easily modify these definitions in order to customise aspects of code generation, and to create new definitions for conversions to other languages.

Due to this standardisation of how conversions are specified, we have created a small repository of CeLEDS/CeLEDSExporter compatible conversion definitions, including definitions for C, MATLAB, and Python. This repository can be found in the CeLEDS/languages subdirectory of the CellML API source code. We have also created a definition for FORTRAN77, although it requires further testing before being considered ready for widespread use.

### The CellML Integration Service

The CellML Integration Service (CIS) API provides an interface for performing simulations of models, and receiving asynchronous notifications as results become available.

The IDL specification for the CIS API can be found in the file interfaces/CIS.idl, in the CellML API source tree.

CellML Model interface pointers are given to CIS, which then creates a CellMLCompiledModel object. The application then specifies the algorithm to be used, and the parameters of the simulation (such as error tolerances, maximum step sizes, and parameters controlling which points are reported back). The application may also choose to override an initial value without recompiling the model.

The IntegrationProgressObserver interface can be implemented by the application, and given to the CellMLIntegrationRun interface prior to starting the simulation. This interface receives information about the values of constants which were computed, as well as the results from each time-step, and an indication of whether the integration has succeeded or failed (with an error message in the latter case).

Our implementation of the CellML API internally makes use of CCGS to generate C code. The C code is then compiled using a compiler. For example, in one of our applications based on the CellML API, we bundle a stripped down version of the C compiler from the Free/Open Source GNU Compiler Collection [33] (gcc) with our application. The code is then linked into a shared object and dynamically loaded into the CIS implementation, which then uses a separate program thread to simulate the model (using either an ODE solver from the SUNDIALS CVODE project [34], or an ODE solver from the GNU Scientific Library [35], depending on the algorithm requested).

### Test-suite

We have also developed an extensive test-suite for validating API implementations. For the core API (including DOM and MathML DOM), and some extension APIs, a program included with the test-suite makes use of every attribute and operation in the API, and checks that invariants which are expected to be true, if the implementation behaves correctly, are in fact true. In addition, the test-suite also includes a series of small programs, as well as a series of inputs to those programs, and expected outputs. For example, the program CellML2C is a small, command-line driven test program, that takes a CellML model as input, and uses the CCGS extension API to generate C code from it. The test-suite calls CellML2C with 17 different models (each of which are crafted to contain peculiarities to test different features). Our API implementation is automatically tested against this test-suite after every commit, on Linux, Mac OS X, and Windows XP, with *ad hoc *testing on a range of other platforms. The API implementation currently passes all of the above tests.

In the future, we plan to add tests which can confirm that the numerical results provided by implementations of the CellML Integration Service are correct, in a similar vein to the SBML test-suite http://sourceforge.net/projects/sbml/files/test-suite/2.0.0%20alpha/.

### Comparison with libSBML

The CellML API is, to our knowledge, the first publicly available API that supports the processing of CellML models. However, there are other similar projects designed to process mathematical models in different encodings.

LibSBML is, in many ways, analogous to the CellML API, except that it processes SBML models. As CellML provides a higher level of domain independence than SBML, it is expected that tools used across many different domains of expertise will need to exchange CellML models. In addition, some tools, such as generic modelling environments, may need to import and export both SBML and CellML models, in which case, both a CellML API implementation, and libSBML can be used together in the same program. Aside from the difference in language support, there are some additional major differences between the CellML API and libSBML.

The CellML API emphasises the technical separation of the interface definition (i.e. the API itself, described using IDL) and implementations of the interface. With the CellML API, adding a new language binding involves developing code to automatically produce a wrapper from the IDL description, rather than using SWIG [36] (which may require this to be followed by manual creation of wrappers to tidy up the details). We therefore expect that the CellML API approach is more robust to changes to the API, and to the addition of completely new modules.

In addition, the CellML API takes a different approach to the manipulation of MathML. The CellML API requires implementations to support an existing API, the MathML DOM [20], while libSBML provides a more limited Abstract Syntax Tree (AST) based approach. The libSBML approach, however, does allow for translations to and from plain text; this feature is available in OpenCell (see below), and we plan to include the feature in a future version of the CellML API.

## Discussion

### Applications of the API

Our CellML API implementation is at the point at which it is stable enough for widespread use. It is already used extensively by the OpenCell [37] environment (formerly known as PCEnv), which provides support for viewing, editing, and running simulations from CellML models. It is also used by the Physiome Model Repository [38].

In addition, third-party users have applied the API to process CellML models and carry out simulations and post-processing [39,40].

### Ongoing support for the API

The CellML API is a Free/Open Source project, and contributions from any interested parties are encouraged. The project is regularly updated to support new features.

A public mailing list has been set up to allow communication amongst developers using and improving on the CellML API and other CellML tools. Users can view the archives and subscribe at http://www.cellml.org/mailman/listinfo/cellml-tools-developers/.

In addition, other development facilities, including an issue tracker for bugs and feature ideas https://tracker.physiomeproject.org, and an automated build and test monitoring system http://autotest.bioeng.auckland.ac.nz/cellml-build/, are provided.

### Future developments

A number of potential future contributions to the API and implementation are under consideration. For example, there is a proposal for a new API for converting CellML models to and from input languages (non-CellML text-based languages that are more easily human readable).

In addition, there are plans to make CCGS support dedicated DAE solvers such as IDA [41], which could allow for DAEs of arbitrary index to be solved.

Another important future improvement is the addition of more language bindings. The choice of language bindings will depend on the input we receive from the community, but could, for instance, include Python, Ruby, and Haskell.

Other important improvements for future consideration include improving the documentation of the API, providing better support for working with metadata, and providing utilities for easier symbolic manipulation of mathematics.

## Conclusions

The CellML API and its implementation are available, and are ready for widespread adoption by the community. Developers of tools which process mathematical models are strongly encouraged to support CellML, so that users of the tool can participate in model sharing, with all the associated benefits to the scientific community. The CellML API and its implementation provide facilities which should make this task substantially easier.

## Availability and requirements

• Project name: The CellML API. Version 1.6 was the latest release at the time of writing.

• Project home page: http://www.cellml.org/tools/api/

• Operating systems: The API implementation can be built on any POSIX like system, including Linux, Mac OS X, and Cygwin. It can also be built using Microsoft Visual C++ 2008. It has been tested on Linux (x86, AMD64, PowerPC), AIX, Windows (XP and Vista) and Mac OS X.

• Programming language: The API is in IDL (language independent), and the implementation in C++, callable through bridges from C++, Java, JavaScript, and from other languages through CORBA.

• Other requirements: The build requires the omniidl tool, which is part of omniORB [42], as well as libxml2 [18], and optionally the GNU Scientific Library (GSL) [35]. If the Java bindings are desired, the Java Development Kit is required. If JavaScript (XPCOM) bindings are desired, XULRunner is required [17].

• License: The CellML API and implementation can be redistributed under any one of: the GNU GPL version 2 or later, the GNU LGPL version 2.1 or later, or the Mozilla Public License version 1.1. This allows the API and implementation to be used in a wide range of public and private research and applied settings.

• Any restrictions to use by non-academics: There are no restrictions on usage of the API. Redistribution requires compliance with one of the licenses above, as well as the licenses of any dependencies being used (for example, if GSL support is enabled, redistribution must be under the terms of the GPL).

The source code and change history is available on SourceForge. Documentation on how to build the API on various platforms is included in the 'docs' directory of the source tree. In addition, the documentation extracted from the IDL files using the Doxygen tool are available in HTML form. Links to these resources can be found on the project home page.

## List of abbreviations used

AnnoTools: The Annotation Tools; API: Application Programming Interface; CCGS: The CellML Code Generation Service; CeLEDS: The CellML Language Export Definition Service; CellML: An XML-based language for describing mathematical models; CeVAS: The CellML Variable Annotation Service; CIS: The CellML Integration Service; CORBA: Common Object Request Broker Architecture; CUSES: The CellML Units Simplification and Expansion Service; DAE: Differential Algebraic Equation; DOM: Document Object Model; IDL: Interface Definition Language; MAL: MathML to Language; MaLaES: The MathML Language Expression Service; ODE: Ordinary differential equation; RDF: Resource Description Format; URI: Uniform Resource Indicator; VACSS: The Validation Against CellML Specification Service; XML: The Extensible Markup Language; XPCOM: Cross-platform Common Object Model.

## Authors' contributions

AKM developed most of the API and its implementation and wrote the first draft of the manuscript. MH contributed to the development of an earlier API, and provided guidance on the development of the API discussed here. JM contributed to the development of the API and implementation. AR developed the CeLEDS and CeLEDSExporter modules. AG contributed documentation for the API. PN, RB, AG, and JC contributed advice on the development of the API and its implementation. All authors provided input into this manuscript.

## References

[B1] Ascher U Petzold L Computer methods for ordinary differential equations and differential-algebraic equations 1998 Society for Industrial Mathematics

[B2] Clancy C Rudy Y Linking a genetic defect to its cellular phenotype in a cardiac arrhythmia Nature 1999 400 6744 566 10.1038/23034 10448858

[B3] Volterra V Variations and fluctuations of the number of individuals in animal species living together ICES Journal of Marine Science 1928 3 3 10.1093/icesjms/3.1.3

[B4] Hunter P Nielsen P A strategy for integrative computational physiology Physiology 2005 20 5 316 325 10.1152/physiol.00022.2005 16174871

[B5] Garny A Nickerson D Cooper J Santos R Miller A McKeever S Nielsen P Hunter P CellML and associated tools and techniques Philosophical Transactions A 2008 366 1878 3017 10.1098/rsta.2008.0094 18579471

[B6] Bray T Paoli J Sperberg-McQueen C Maler E Yergeau F Extensible markup language (XML) 1.0 W3C recommendation 2000

[B7] Lloyd C Lawson J Hunter P Nielsen P The CellML model repository Bioinformatics 2008 24 18 2122 10.1093/bioinformatics/btn390 18658182

[B8] Le Novere N Bornstein B Broicher A Courtot M Donizelli M Dharuri H Li L Sauro H Schilstra M Shapiro B BioModels Database: a free, centralized database of curated, published, quantitative kinetic models of biochemical and cellular systems Nucleic Acids Research 2006 34 Database D689 10.1093/nar/gkj092 16381960 PMC1347454

[B9] Hucka M Finney A Sauro H Bolouri H Doyle J Kitano H The systems biology markup language (SBML): a medium for representation and exchange of biochemical network models Bioinformatics 2003 19 4 524 10.1093/bioinformatics/btg015 12611808

[B10] Bornstein B Keating S Jouraku A Hucka M LibSBML: an API Library for SBML Bioinformatics 2008 24 6 880 10.1093/bioinformatics/btn051 18252737 PMC2517632

[B11] Siegel J OMG overview: CORBA and the OMA in enterprise computing 1998

[B12] Hedley W Nelson M CellML 1.0 Specification 2001 http://www.cellml.org/specifications/cellml_1.0 10.2390/biecoll-jib-2015-259 26528557

[B13] Cuellar A Nielsen P Halstead M Bullivant D Nickerson D Hedley W Nelson M Lloyd C CellML 1.1 Specification 2002 http://www.cellml.org/specifications/cellml_1.0

[B14] Doxygen developers Doxygen Manual http://www.stack.nl/~dimitri/doxygen/manual.html

[B15] Koenig A The C++ Language Standard. Report ISO/IEC 14882: 1998

[B16] Gosling J Joy B Steele G Bracha G The Java (TM) Language Specification 2005 Addison-Wesley Professional

[B17] Stearn B XULRunner: A New Approach for Developing Rich Internet Applications IEEE Internet Computing 2007 67 73 10.1109/MIC.2007.75

[B18] Veillard D Libxml2: The XML C parser and toolkit of Gnome

[B19] Wood L Programming the Web: the W3C DOM specification IEEE Internet Computing 1999 3 48 54 10.1109/4236.747321

[B20] Carlisle D Ion P Miner R Poppelier N Mathematical markup language (mathml) version 2.0 W3C Recommendation 2001 21

[B21] Lutz M Programming python 2006 O'Reilly Media, Inc

[B22] Bray T Hollander D Layman A Namespaces in XML W3C recommendation 1999

[B23] Beard D Britten R Cooling M Garny A Halstead M Hunter P Lawson J Lloyd C Marsh J Miller A CellML metadata standards, associated tools and repositories Philosophical Transactions A 2009 367 1895 1845 10.1098/rsta.2008.0310 PMC3268215 19380315

[B24] Beckett D McBride B RDF/XML syntax specification (revised) W3C Recommendation 2004 10

[B25] Klyne G Carroll J McBride B Resource description framework (RDF): Concepts and abstract syntax W3C recommendation 2004 10

[B26] Bevan D Distributed garbage collection using reference counting Volume II: Parallel Languages on PARLE: Parallel Architectures and Languages Europe table of contents 1987 176 187

[B27] Stroustrup B Exception safety: concepts and techniques Lecture notes in computer science 2001 60 76 full_text

[B28] Tarjan R Efficiency of a good but not linear set union algorithm J Assoc Comput Mach 1975 22 215 225

[B29] Bureau International des Poids et Mesures The International System of Units (SI), 8th edition 2006

[B30] Hairer E Wanner G Solving Ordinary Differential Equations. II. Stiff and Differential-Algebraic Problems. 1996 Springer Ser Comput Math 1996

[B31] Lambert J Numerical methods for ordinary differential systems: the initial value problem 1991 John Wiley & Sons, Inc. New York, NY, USA

[B32] Kernighan B Ritchie D Ejeklint P The C programming language 1988 Prentice-Hall Englewood Cliffs, NJ

[B33] Stallman R Using GCC: The GNU Compiler Collection Reference Manual 2003

[B34] Cohen S Hindmarsh A CVODE, a stiff/nonstiff ODE solver in C Computers in physics 1996 10 2 138 143

[B35] Galassi M Davies J Theiler J Gough B Jungman G Booth M Rossi F GSL-GNU Scientific Library: Reference manual 200415554402

[B36] Beazley D SWIG: An easy to use tool for integrating scripting languages with C and C++ Proceedings of the 4th conference on USENIX Tcl/Tk Workshop, 1996-Volume 4, USENIX Association 1996 15

[B37] OpenCell developers OpenCell software package http://www.opencellproject.org/.

[B38] Tommy Yu The Physiome Model Repository software package http://www.cellml.org/tools/pmr

[B39] Nickerson D Buist M Practical application of CellML 1.1: The integration of new mechanisms into a human ventricular myocyte model Progress in Biophysics and Molecular Biology 2008 98 38 51 10.1016/j.pbiomolbio.2008.05.006 18606438

[B40] Nickerson D Corrias A Buist M Reference descriptions of cellular electrophysiology models Bioinformatics 2008 24 8 1112 10.1093/bioinformatics/btn080 18310619

[B41] Hindmarsh A Brown P Grant K Lee S Serban R Shumaker D Woodward C SUNDIALS: Suite of nonlinear and differential/algebraic equation solvers ACM Transactions on Mathematical Software (TOMS) 2005 31 3 396

[B42] Grisby D Lo S Riddoch D The omniORB version 4.0 User's Guide3212140

[B43] Kobryn C Modeling components and frameworks with UML Communications of the ACM 2000 43 10 31 38 10.1145/352183.352199

